# Additive Manufacturing of Polymer/Bioactive Glass Scaffolds for Regenerative Medicine: A Review

**DOI:** 10.3390/polym15112473

**Published:** 2023-05-26

**Authors:** Andrea Martelli, Devis Bellucci, Valeria Cannillo

**Affiliations:** Dipartimento di Ingegneria Enzo Ferrari, Università degli Studi di Modena e Reggio Emilia, Via. P. Vivarelli 10, 41125 Modena, Italy; andrea.martelli@unimore.it

**Keywords:** additive manufacturing, polymer, bioactive glass, composite, scaffold, tissue engineering

## Abstract

Tissue engineering (TE) is a branch of regenerative medicine with enormous potential to regenerate damaged tissues using synthetic grafts such as scaffolds. Polymers and bioactive glasses (BGs) are popular materials for scaffold production because of their tunable properties and ability to interact with the body for effective tissue regeneration. Due to their composition and amorphous structure, BGs possess a significant affinity with the recipient’s tissue. Additive manufacturing (AM), a method that allows the creation of complex shapes and internal structures, is a promising approach for scaffold production. However, despite the promising results obtained so far, several challenges remain in the field of TE. One critical area for improvement is tailoring the mechanical properties of scaffolds to meet specific tissue requirements. In addition, achieving improved cell viability and controlled degradation of scaffolds is necessary to ensure successful tissue regeneration. This review provides a critical summary of the potential and limitations of polymer/BG scaffold production via AM covering extrusion-, lithography-, and laser-based 3D-printing techniques. The review highlights the importance of addressing the current challenges in TE to develop effective and reliable strategies for tissue regeneration.

## 1. Introduction

In recent decades, there has been a general ageing of the world population that is associated with an increase in age-related pathologies. The advancement of science and technology has made it possible to replace diseased tissues of the human body with grafts in order to restore normal biological functions. In general, grafts can be distinguished depending on their origin. Autografts, in which the patient’s own tissues are used, are considered the gold standard for grafting procedures. Autografts provide osteoinductive growth factors, osteogenic cells, and an osteoconductive structure. However, autografts have limits regarding donor site morbidity and availability. These limitations have been overcome by allografts, in which the patient and the donor are not the same person. Therefore, allografts introduce the risk of disease transmission and rejection [1,2]. Similarly, xenografts, in which the donor belongs to another species such as pigs or cows [3], could be unsuccessful due to a vigorous rejection response [4]. Thanks to their origin, synthetic grafts eliminate the issues related to immunological factors, although a complete biological outcome has not yet been obtained [1,2].

The science involved in replacing or regenerating human cells, tissue, or organs to restore or establish normal function is called regenerative medicine (RM) as stated by Mason et al. [5]. In particular, the branch of RM that deals with studying, designing, and producing synthetic grafts is tissue engineering (TE). There are two approaches to TE that can be used to regenerate damaged tissue: ex vivo and in situ. In ex vivo TE, cells and biomolecules are combined with temporary, highly porous scaffolds outside the body to create tissue constructs that can be implanted [6]. While this approach can mimic native tissue functions, it has several limitations. These include donor-tissue morbidity, the need for large quantities of immune-acceptable cells, and challenges associated with in vitro cell expansion. In addition, it can be difficult to replicate autocrine and paracrine signalling effects. In situ TE, on the other hand, leverages the body’s natural ability to regenerate tissue, eliminating the need for ex vivo cell manipulation. There are several in situ TE approaches; these include the use of biomaterials loaded with bioactive cues to guide functional restoration to the site of injury [7]. These methods are relatively simple, reduce regulatory hurdles, and do not require complex cell culture conditions to obtain functional tissues. Furthermore, the shelf life of synthetic scaffolds is longer than that of cell-laden scaffolds, making in situ approaches more favorable for clinical translation [7].

**Figure 1:**
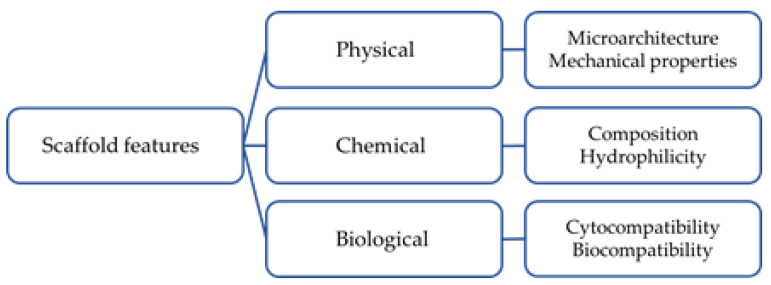
Schematic representation of scaffold features and requirements.

As mentioned before, one of the means available to TE are scaffolds. Scaffolds provide a 3D porous structure for the regeneration of damaged tissue; for example, in critical-sized defects. In fact, such defects cannot be expected to heal without additional intervention. Even if the dimensions and size of a critical-sized defect depend on the specific location within the body and various patient-related factors, they are usually defined as bone voids that are larger than twice the diameter of the affected bone or involve more than 50% circumferential bone loss [8,9]. Scaffolds mimic the functions of the native extracellular matrix (ECM) in human tissue, which provides a supportive environment for cells to attach, migrate, and differentiate to create new tissue [10]. Moreover, scaffolds are designed to mimic the shape, microstructure, and mechanical properties of the damaged tissue, which affect effective tissue regeneration. The scaffold’s porosity and surface properties must be optimized to facilitate cell attachment, migration, and differentiation [10,11]. Additionally, scaffolds must satisfy some features (as summarized in Figure 1) to be safe and promote tissue regeneration; these include cyto- and tissue compatibility, mechanical, architecture properties, and bioactivity [10,12]. Scaffolds are considered bioactive when they can achieve a controlled interaction and reaction with the surrounding tissue in a physiological environment [13]. Moreover, scaffolds can be used as a delivery vehicle for bioactive molecules (e.g., drugs, antibiotics, etc.) or to contain cells [14,15,16,17]. Finally, scaffolds can be realized with different materials (e.g., metals, polymers, ceramics, and composites) and by means of different protocols that obtain disparate results [18]. 

Ceramic implants have excellent bioactivity and biocompatibility, meaning they are capable of interacting favorably with the body and eliciting an appropriate physiological response in specific applications [19]. However, ceramics are brittle, which is in contrast to polymers and metallic implants that have higher mechanical properties but lower bioactivity and biocompatibility. To combine the best properties of both ceramics and other materials, two alternative approaches have emerged in the last two decades. The first approach involves using a bioceramic scaffold as a matrix and coating it with polymer [20,21]. The second approach involves adding bioceramics as a coating or filler to a polymer matrix [20]. This method has led to the development of a new generation of biomaterials that are composite materials [22,23] that properly benefit from unique properties of their constituents.

Among bioceramics, bioactive glasses (BGs) have gained a lot of attention since Hench’s discovery of 45S5 Bioglass^®^ in the 1970s [24]. Thanks to their composition and amorphous nature, BGs can easily interact with the receiver body. The mechanism of bonding to living bone tissue involve a sequence of 12 steps. The first five stages occur quickly on the surface due to fast ion exchange, network dissolution, silica-gel polymerization, and chemisorption. These processes lead to the crystallization of a surface layer of hydroxyapatite (HA), which is highly bioactive due to its composition and structure resembling that of biological apatite (the mineral phase of bone). Subsequent steps including the adsorption of growth factors and a synchronized sequence of cellular events result in the formation of new bone tissue [25]. BGs can be classified into three categories based on their composition: silicate, borate/borosilicate, and phosphate glasses. The difference is in the network former (Si^4+^, B^3+^, and P^5+^, respectively) responsible for forming the 3D network [26]. As the network former varies, the properties of the glass also vary. In particular, borate glasses have higher reaction and conversion rates into HA than their silica counterparts [27,28]. To expand the working window of BGs (the temperature difference between the glass transition temperature and the crystallization temperature that allows sintering without crystallization [29]), various new compositions have been developed through studies over the years; e.g., 13-93, ICIE6, and BGMS10 [30,31,32]. Additionally, doping BGs with different ions can also result in improved angiogenesis or osteogenesis. This process modifies the biological response of BGs, leading to enhanced properties for specific applications [33,34,35,36,37,38,39].

On the other hand, polymers can be categorized into two main types: natural and synthetic. Examples of natural polymers include collagen, gelatin, and alginates, which are known for their good to excellent biocompatibility and biodegradability but often have poorer mechanical properties compared to synthetic polymers. Some commonly used synthetic polymers in TE include polycaprolactone (PCL), polylactic acid (PLA), and polyethylene glycol (PEG) [40]. These materials offer a wide range of mechanical and degradation properties that can be tailored for specific tissue engineering applications. Moreover, some polymers can be photosensitive, meaning their physical or chemical properties can change upon exposure to light [41]. Photopolymers, unlike thermoplastic polymers, have a cross-linked molecular structure that does not melt and exhibit much less creep and stress relaxation [42]. This property allows the production of scaffolds via lithography, which is a promising technique of additive manufacturing (AM) that will be covered in depth in the following paragraphs.

The combination of BGs’ bioactivity and the versatility of polymers creates a strong potential for polymer/BGs scaffolds to achieve exceptional regeneration rates compared to monomaterial scaffolds [43]. As a result, there is significant interest in and ongoing research aimed at developing and optimizing these composite materials.

As previously mentioned, scaffolds can be created with different methods. Conventional protocols for scaffold production are defined as processes that result in a homogenous, continuous pore structure without any long-range channeling microarchitecture [44]. This nonspecific microarchitecture leads to limitations in terms of finely controlling the geometry, pore size, and pore interconnectivity of the scaffold structure [45]. One of the most representative examples of conventional methods is the foam replica method. This method involves replicating the porous structure of a sacrificial template to obtain a positive replica composed of glass or glass–ceramic particles, which are then sintered around the organic strut. This method is versatile because it allows for the use of a wide variety of sacrificial templates of both synthetic and natural origin. It is also relatively inexpensive and requires no special equipment [46]. However, this technique has some limitations, including a lack of reproducibility and control of the architecture in large or complex samples due to the formation of slurry gradients and pore occlusion in a confined portion of the volume, resulting in variable porosity between different samples [47]. As a result, the operator’s skill level strongly influences the final outcome, making it difficult to standardize the manufacturing process [46]. Moreover, it is not possible to achieve pore architecture and porosity tailored to a specific patient’s needs [48]. The limitations of conventional manufacturing methods have been overcome using AM, which is a layer-by-layer deposition process that can be performed with or without a substrate. Compared to traditional methods, AM offers improved material utilization, reduced waste, and greater design freedom [49]. AM is based on advanced computer modeling, enabling the creation of complex shapes and internal structures. This capability allows for the production of scaffolds with precise control over their hierarchical pore structure, which can be tailored to meet the specific needs of individual patients [45]. Moreover, bioprinting (a subcategory of AM) enables the printing of structures using viable cells and biological molecules. This approach addresses the issue of cell homogeneity, resulting in faster integration with the host tissue, lower risk of rejection, and most importantly, uniform tissue growth in vivo [50].

Many reviews regarding the AM technique for scaffolding have been written, but most of them focused on a specific tissue or material [45,51,52,53]. In particular, just a few authors have discussed scaffolds containing BGs; for example, Dukle et al. highlighted the bottlenecks in 3D printing of BGs [54], while Simorgh et al. reviewed the application of BGs for the fabrication of 3D-printed and bioprinted scaffolds and their usability in TE [55]. Therefore, the aim of this review was to provide an overview of the main AM techniques able to produce polymeric composite scaffolds containing BGs while emphasizing the potentials and limitations of each specific protocol. 

## 2. Extrusion-Based 3D Printing

Extrusion-based 3D printing involves depositing layers of materials such as polymers onto a baseplate using a movable nozzle. The feedstock can come in various forms, including filaments, granules, or pastes [54]. Examples of this technology include fused deposition modeling (FDM), powder extrusion deposition (PED), and direct ink writing (DIW). 

### 2.1. Fused Deposition Modeling

Fused deposition modeling (FDM) is a method based on the extrusion of a polymer through a nozzle movable in X–Y–Z onto a base plate. The resolution of the printer is defined by the dimensions of the nozzle and the precision of its movement control. The choice of polymeric material should meet two main criteria: a relatively low melting temperature and an appropriately high glass transition temperature [56]. The typical extrusion temperature range is 100–140 °C [57]. The polymer can be mixed with BGs and extruded to obtain a filament used in FDM. This approach offers some advantages over the paste-based approach because it is a solvent-free method that allows the material to be printed with a pore size gradient [58]. However, the extrusion temperatures are too high to include cells or bioactive molecules [59]. PCL is widely used in scaffold production (particularly through FDM) due to its low melting point (59–64 °C), good drug permeability, biocompatibility, solubility, excellent compatibility in blends, and ability to maintain its mechanical and physical properties long enough for tissue growth [60]. 

Wang et al. [61] produced PCL/58S scaffolds with varying percentages of BG content (0, 5, 10, and 20 wt%). 58S is a silicate BG produced via sol–gel processing designed to eliminate Na_2_O from its composition [62]. In vitro and in vivo studies demonstrated that the scaffold with the highest BG content exhibited the most profound bone repair effect, exhibiting a maximum compressive strength of 43.52 ± 2.01 MPa and a minimum tensile strength of 4.63 ± 0.25 MPa. Despite these favorable outcomes, the best contact angle achieved was 87.99° ± 7.07°, indicating that the hydrophilicity of the scaffold was not optimal for cell adhesion. As is known, the adhesion of cells to artificial materials depends on several surface properties including wettability, roughness, surface charge, and chemical functionalities [63]. Cells were found to adhere effectively to surfaces with moderate wettability and water contact angles in the range of 40–60° (as reported by Arima et al. [64]). Ilyas et al. [65] successfully addressed this issue by altering the surface of scaffolds and achieving water contact angles between 30 and 65°. Such modification not only improved cell adhesion but also conferred enhanced antibacterial properties. The study involved the creation of composite PCL scaffolds incorporating either silicate or borate BG particles (1393 and 1393-B3 glasses) coated with Sr-doped mesoporous bioactive glass nanoparticles and gelatin and further modified with polydopamine (PDA). 13-93 is a silica-based BG that contains K_2_O and MgO to expand its working range [30]. A modified version of 13-93 known as 13-93B3 was obtained by replacing all the SiO_2_ in 13-93 with B_2_O_3_, which led to a higher degradation rate [66]. SEM analysis showed a uniform distribution of BG particles thanks to the hydrophobic nature of PCL. The scaffolds containing borate BG particles demonstrated equivalent or even better cell viability compared to those with silicate BG particles. An alternative approach for altering the surface of scaffolds was found by Fathi et al. [67] by utilizing NaOH, which effectively decreased the contact angle of the scaffolds from 76° to 61°. The study investigated several compositions of BG doped with Sr, Co, or both. The aim of adding dopants was to promote both osteogenesis and angiogenesis. The most optimal mechanical properties were observed in the composition that contained both Sr and Co dopants, exhibiting a Young’s modulus of over 9 MPa. Kim et al. [68] identified a limit on the BG content for PCL scaffolds produced by FDM. They discovered that a BG content exceeding 60% led to high viscosity, hindering the effectiveness of the FDM process. Through their study of different ratios of PCL to BGS-7 (for further information about this BG, please refer to [63]), they identified that a BG content of 40 wt% represented the best compromise between mechanical stability and biological compatibility.

Another polymer widely used in FDM is PLA due to its biocompatibility, biodegradability, and suitable mechanical properties. Moreover, the degradation rate as well as the physical and mechanical properties of PLA can be adjusted by varying the molecular weight [69,70]. In their initial attempt, Distler et al. [58] created filaments using PLA with varying amounts of 45S5 (0, 1, 2.5, 5, and 10 wt%). However, the bonding between the materials was poor, which resulted in suboptimal mechanical properties even at a high BG content. To improve interface adhesion, the authors suggested exploring alternative surface modifications (e.g., particle roughness, size, and chemistry) and adjusting the bulk polymer chemistry to enhance polymer/filler interface binding. Saranti et al. [71] did not address the bonding issue between PLA and 45S5 in their study. Instead, they fabricated PLA scaffolds with varying amounts of 45S5 and incorporated biocompatible, fluorescent carbon dots (C-dots) to enable real-time monitoring of the healing process. C-dots are biocompatible and fluorescent nanoparticles with sizes up to 10 nm that exhibit notable photophysical and chemical properties. The C-dots used were red-emissive; these are preferred for biomedical applications because they operate in the near-infrared (NIR) region, reducing tissue absorption and autofluorescence [72]. In vitro cytotoxicity tests indicated that the composites produced in their study could be safely used in vivo at low concentrations (<20 ppm). 

FDM technology is capable of printing biopolymers such as sodium alginate (SA). Luo et al. [73] developed an organic–inorganic ink by blending 13-93 BG/SA with polyvinyl alcohol (PVA) in different ratios, resulting in scaffolds with improved mechanical properties when compared to pure SA scaffolds. The samples showed increased HA mineralization and released Mg^2+^ and SiO^4−^ ions. The best results were obtained for a BG/SA ratio of 2:4 with a compressive strength of 16.74 ± 1.78 MPa, a modulus of 79.49 ± 7.38 MPa, and the highest activity of primary rat bone mesenchymal stem cells (rBMSCs).

In summary, the FDM process allows the production of complex 3D structures with resolution limited by the nozzle diameter, the layer height, and the viscosity of the polymer [57]. Cells or bioactive molecules cannot be included during the printing process due to the high extrusion temperature, but they can be added after printing. FDM enables the production of parts with tuneable mechanical properties using a wide range of materials, including those with BG content up to 60 wt%. 

### 2.2. Precision Extrusion Deposition

Precision extrusion deposition (PED) is a variant of FDM that allows printing directly from granules without the need to create filaments. Extrusion is obtained with a screw, allowing an accurate control on the quantity extruded. Moreover, the XY motion system is synchronized with the extruder, obtaining precise control of the thickness and position of the fibers [74,75,76]. In addition, it is possible to use a wider range of biopolymers with higher melting point by installing an assisting cooling device near the nozzle of the PED [76].

Limited research exists on PED-produced composite scaffolds. Petretta et al. [77] produced PCL/BG composite scaffolds with different ratios of PCL to BG (70/30 and 50/50) and found that the 50/50 ratio yielded better results with a compressive modulus within the range required for trabecular bone (0.1–5 GPa). Furthermore, cytotoxicity tests showed no adverse effects for either ratio up to day 21. Bioactivity tests revealed that the 50/50 composition exhibited a superior performance compared to the 70/30 ratio. This result may be attributed to the greater amount of BG that emerged from the polymer matrix in the 50/50 ratio, resulting in an enhanced surface roughness that promoted cell proliferation. A similar compressive modulus was found by Daskalakis et al. [78] when producing PCL-based composite scaffolds with different concentrations of 45S5 (0–10–15–20 wt%). Consistent with previous studies, increasing the BG content led to improved mechanical performance, while the hydrophilic behavior was not significantly affected by the addition of BG. Interestingly, the scaffold with 10% BG exhibited the best cell activity at day 14 after cell seeding. These studies suggested that PED is a promising method for producing scaffolds containing BGs. However, the low mechanical properties of the scaffolds indicate that the technique is not yet mature enough for loading applications.

### 2.3. Direct Ink Writing

Direct ink writing (DIW), also known as robocasting, is an extrusion-based layer-by-layer process that employs highly concentrated powder suspensions (slurries) composed of a mixture of ceramic powder (>40 vol%), water, and additives (<3 vol%) such as dispersant, binders, and coagulants [79]. To be suitable as ink for robocasting manufacturing, a colloidal slurry should possess several key properties. Firstly, it should exhibit a rheology that is “yield pseudoplastic”, meaning that the viscosity decreases as the applied stress increases. Secondly, it should be able to flow smoothly through a small nozzle. Finally, it should have the ability to maintain its shape after deposition [79]. When these requirements are satisfied, DIW can be employed to fabricate intricate composite bodies that sinter into relatively strong, dense, and defect-free parts. It is important to note that the resulting product obtained through DIW is transformed into a monomaterial and is no longer a composite [80]. The range of printing parameters and materials used for DIW were reviewed by Balani et al. [81]. Among all binders, Pluronic F-127 has been shown to be a universal binder for different types of BGs regardless of their reactivity and composition [82]. Pluronic F-127 is an amphiphilic triblock copolymer composed of a central hydrophobic chain of poly(propylene oxide) flanked by two hydrophilic chains of poly(ethylene oxide). This composition makes Pluronic F-127 a water-soluble surfactant with rheological behavior that can be thermally reversed [83]. In the same study on Pluronic F-127, Nommeots-Nomm et al. [82] evaluated the sintering outcomes of two silicate bioactive glasses with low-silica content (PSrBG and ICIE16) in comparison to 13-93 bioactive glass. To preserve the amorphous structure of the glass, the researchers used a large particle size in their samples, but this led to a limited compressive strength after sintering that ranged between 32 and 48 MPa. Despite these limitations, the investigated compositions displayed satisfactory sintering outcomes with uniform shrinkage and a porosity between 41 and 43 vol%. This finding suggested that the sintering outcomes of low-silica content BGs can be improved with larger particle sizes. In addition, Ben-Arfa et al. [84] produced scaffolds using a high-silica sol–gel glass (HSSGG) via DIW to improve sintering and densification. However, during the thermal treatment, the BG powders experienced partial crystallization, leading to limited densification and to a scaffold with 36% open microporosity. Despite the improved responsiveness of the samples both in vitro and in vivo, this limited their compressive strength to 5 MPa. To address this issue, the authors added copper or lanthanum to the HSSGG, which had minimal effects on ink printability but significantly improved the mechanical properties of the final products. In particular, the Cu-doped glass scaffold showed a compressive strength even higher than human trabecular bone. In vitro tests confirmed the BGs’ biocompatibility with respect to various cell lines, making them suitable for in vivo experimentation [85,86]. The sintering of 45S5 scaffolds can be challenging due to the small sintering window [29]. Motealleh et al. [87] have extensively studied the use of DIW to produce amorphous 45S5 scaffolds. The amorphous scaffolds exhibited a faster degradation in simulated body fluid (SBF) compared to 45S5 crystallized scaffolds, resulting in a more rapid decrease in mechanical properties. Additionally, cell tests suggested that crystallized scaffolds had an improved cell viability. To enhance the compressive strength of the amorphous scaffolds, Motealleh et al. [88] coated them with an HA/PCL nanocomposite coating. The study showed that the coated scaffolds had improved mechanical properties by more than 200% but did not provide information about the biological response of the coated scaffolds. In order to promote cell adhesion and bone regeneration, a given scaffold must exhibit a hierarchical structure with pores of different sizes. To achieve this, Barberi et al. [89] produced BG scaffolds with a porosity gradient using Pluronic F-127 as the binder. However, the scaffolds obtained had defects such as voids and cracks due to air bubbles entrapped in the ink as well as different shrinkage rates between the denser core and more porous outer shell. Despite these issues, mechanical tests showed that the compressive strength of the samples was comparable to the range of human trabecular bone. However, the values exhibited a great variability. The issue of the defects and the variability in mechanical properties of scaffolds with a porosity gradient is still a challenge that needs to be addressed. To overcome these challenges, Touré et al. [90] developed a novel approach by combining DIW and electrospinning to fabricate composite scaffolds with multigrade porosity. They evaluated a mixture of PCL and poly(glycerol sebacate) (PGS) with varying concentrations of BGs at 0, 5, and 10 wt%. The hybrid scaffold fabrication method was effective in achieving excellent adhesion between the 3D-printed layer and the electrospun network, which improved the stiffness of the scaffolds with a 2.5-fold increase in the Young’s modulus. While the addition of BGs only slightly affected the mechanical response of the scaffolds (a 30% increase in elastic modulus for scaffolds with 10 wt% BGs), it significantly impacted the material degradation in vitro and balanced the acidic nature of the PGS. This positively affected the growth of fibroblasts—scaffolds containing 10 wt% BGs sustained the cell viability longer in vitro. Touré et al. recognized the potential application of these composite scaffolds in tendon and ligament replacements in light of their ability to meet the mechanical requirements of native tissues with an elastic modulus in the range of 100–300 MPa. To better understand the degradation rate of silica and borate glasses, Deliormanli et al. [91] conducted a study comparing scaffolds containing 13-93 and 13-93B glass; the maximum BG content that maintained the flowability behavior of the polymeric suspension was used. An X-ray diffraction (XRD) analysis indicated that no crystallization had occurred in the scaffolds. After immersing the scaffolds in SBF for 50 days, mechanical tests revealed that the borate-based scaffolds had a faster decrease in compressive strength (from 65 ± 11 MPa to 8 ± 4 MPa) compared to the silicate-based scaffolds (from 142 ± 20 MPa to 79 ± 32 MPa). This finding demonstrated the superior stability of silicate-based scaffolds in SBF, which makes them promising for the regeneration of load-bearing bones. The influence of silicate BG structure in vivo was investigated by Tulyaganov et al. [92]. The study compared granules and porous scaffolds implanted in rabbit femurs and found no statistically significant difference in new bone formation between the two approaches. However, the scaffold implants resulted in more homogenous tissue formation, underlining the value of this structure. Furthermore, Liu et al. [93] reported an intriguing result for the 13-93 scaffold implanted in rats. They found that the brittle mechanical response of the scaffolds in vitro changed to an elastoplastic response after implantation for longer than 2–4 weeks in vivo, thus highlighting the importance of in vivo testing for the evaluation of scaffold performance. The DIW technique has been expanded to include natural polymers as demonstrated by Dorj et al. [94] in their successful production of a composite scaffold consisting of chitosan and BGs. Chitosan is a semi-crystalline polysaccharide derived from chitin, which is the second most abundant biopolymer after cellulose. Due to its high positive charge, chitosan is often used as a drug carrier [95]. However, it is challenging to solidify chitosan at ambient conditions (as reported by Dorj et al. [94]). To overcome this, the authors used dry ice to refrigerate the environment during printing. The addition of BGs with diameters of a few hundred nanometres and a printing temperature of around −50 °C resulted in a microporous structure. In vitro tests confirmed the scaffold’s ability to form apatite in SBF as well as its cell adhesion and proliferation abilities. However, further research into the scaffold’s mechanical properties is necessary before considering its application. Another promising natural polymer for producing composite scaffolds is silk fibroin (SF) obtained from silkworm cocoons, which exhibits excellent biocompatibility and mechanical properties [96]. Moreover, SF can be manipulated at a temperature close to human physiological temperature, allowing for cell or biological factor loading [97]. Du et al. [98] compared SF/BG and PCL/BG scaffolds (both with a mass ratio of 20/80) and found that the SF/BG scaffold exhibited higher mechanical properties and better biological responses in vitro. In addition, implantation of the scaffolds into the back of nude mice demonstrated the SF/BG scaffold’s more favorable osteogenic ability in vivo. Therefore, SF has great potential as a natural polymer for producing composite scaffolds. When a sintering treatment is not required, DIW allows the printing of scaffolds containing cells or drugs. Kolan et al. [99] developed a method to improve the mechanical properties of hydrogel-based bioinks by modifying a DIW printer to print PLA/13-93B3 and bioink simultaneously. The resulting composite scaffold contained cells and demonstrated enhanced mechanical properties. However, live/dead evaluation revealed nonuniform cell viability with fewer cells surviving in the bottom layer due to a hypoxic-like environment. Wu et al. [100] produced a PVA-based scaffold containing mesoporous BG and dexamethasone (DEX). DEX is a synthetic glucocorticoid that has been shown to promote osteoblast differentiation and bone tissue regeneration. Moreover, DEX has been found to reduce implant-associated inflammation. However, it should be noted that high concentrations of DEX can suppress the proliferation of osteoblasts and result in toxic side effects [101,102,103]. The study found that DEX release was highest after 2 days with slower kinetics up to 10 days, indicating the potential of the scaffold to treat the inflammatory response following implantation. These findings were in agreement with Zhang et al.’s [104] study, which also reported an enhanced osteogenic expression in a Sr-doped BG/PVA scaffold compared to an undoped scaffold.

In summary, DIW allows for the production of scaffolds with several BG compositions, effectively overcoming the sintering difficulties. However, the challenge of fine-tuning the microstructure and mechanical properties remains. To address the mechanical issue, one potential solution could be to coat the samples. However, it is necessary to perform in vivo validation to determine the efficacy of this solution. Further research is needed to address these challenges and fully realize the potential of DIW for scaffold production.

## 3. Lithography-Based 3D Printing

Lithography-based techniques use selective exposure to light to cure a liquid formulation via photopolymerization. The process is initiated by the excitation of a photoinitiator through the absorption of light, which then generates free radicals that start the polymerization process. The process results in the consumption of low-molecular-weight monomers, forming long polymer chains or a polymer network and leading to the solidification of the liquid formulation [86]. Lithography-based techniques are stereolithography and digital light processing.

### 3.1. Stereolithography

Stereolithography (SLA) employs a layer-by-layer deposition of a liquid resin that solidifies via photopolymerization. To further enhance the mechanical properties of SLA-printed objects, a dual-curing process can be employed; e.g., combining photocuring with subsequent thermal curing [105]. Additionally, the feedstock must be a liquid that rapidly solidifies upon illumination, which is a major limitation due to the limited availability of materials that meet this criterion. Creating polymer–ceramic composites requires uniform suspension of ceramic particles in the resin, which affects the slurry viscosity, polymer photoreactivity, and printing resolution [106,107]. SLA’s resolution decreases with increasing print speed [53]; the highest resolution for additive processing is 20 µm, although stereolithography setups based on two-photon absorption can achieve resolutions down to 100 nm [108]. Furthermore, it is possible to incorporate bioactive molecules and cells into the printed structures [109].

One disadvantage of using a low-resolution technique to produce composite scaffolds is that the surface of the scaffold often ends up with a polymer film that covers the BG particles during fabrication. This issue can be resolved by using SLA as demonstrated by Elomaa et al. [110]. In their study on PCL/S53P4 (a BG known for its antibacterial properties that is commercially available as BonAlive^®^ [111]), they showed that SLA allowed for greater control over the surface of the scaffold, resulting in better exposure of the BG particles. Additionally, their study highlighted the variability in mechanical properties between dry and wet scaffolds, which is a crucial consideration for in vivo applications. Understanding this variability is essential to ensure optimal performance and biocompatibility of a scaffold in vivo. Many studies have examined how the composition of suspensions affects the final product. In one such study, Chen et al. [112] investigated the impact of the concentrations of monomers, a reactive diluent, photoinitiator/co-initiator (PI), nonreactive diluent/dispersant, and light absorber on resin rheology and cure depth to optimize the resin for the manufacturing of porous ceramic scaffolds. The researchers found that adding polyethylene glycol 200 (PEG-200) to the suspension improved its stability and shelf life, resulting in a more homogeneous microstructure and increased mechanical strength. However, the hardness value decreased with increasing amounts of PEG-200 because it acted as a plasticizer and lowered the Young’s modulus. The team selected a 10% weight fraction of PEG-200 to avoid changes in mechanical properties. The optimized BG resin composition contained 55% BG, 10% PEG-200 as a nonreactive diluent and rheology modifier, 1% PI, and 0.015% Sudan orange G dye as a light absorber. The SEM and micro-computed tomography (µ-CT) results demonstrated that this composition produced dense structures without cracks. Nevertheless, this composition was evaluated only from a structural perspective, and further research is needed to assess its in vitro and in vivo effects. Kang et al. [113] conducted a study on the optimization of the ceramic suspension of 45S5 for use in commercial 3D SLA printers. They investigated the effect of five different ratios of photocurable resin and acrylate binder and found that the highest reactivity and cure depth were obtained for a 4:6 ratio. Then, they evaluated the effect of varying the 45S5 content in the suspension (ranging from 32% to 40% volume). The maximum strength and viscosity were achieved with a 40% volume fraction of 45S5. Importantly, no residual effects of the binder were found after sintering, suggesting that the suspension with 40% volume fraction of 45S5 can be effectively employed in scaffold manufacturing. The relationship between structural design, pore dimensions, and sintering conditions on mechanical properties in 45S5-based scaffolds was evaluated by Thavornyutikarn et al. [114]. Different microarchitecture and pore sizes were considered. To minimize shrinkage and internal stresses in the scaffold with a pore size of 550 μm, a two-stage sintering process was explored. The study found that partial presintering at 550 °C followed by regrinding and SLA processing and then a final sintering at 950 °C led to an increase in the scaffold’s mechanical strength and a shrinkage reduction. However, this process also caused a reduction in the pore size and porosity that could lead to a reduced cell viability.

In summary, SLA is a 3D-printing process that can work with limited polymers and can also incorporate cells or biomolecules into the feedstock. It can achieve the highest resolution for additive manufacturing processes, although it is generally slower than other methods. However, there is currently limited research on the application of SLA for producing composite scaffolds, and the lack of in vitro and in vivo testing prevents it from being recognized as a suitable method for composite scaffold production at this time.

### 3.2. Digital Light Processing

Digital light processing (DLP) is an advancement of SLA that utilizes a digital micromirror device (DMD) to print the entire layer at once, reducing the time required to produce scaffolds [115]. Like SLA, photopolymerization of suspensions containing ceramic particles depends on the solid content, the powder size, and a refractive index mismatch between the ceramic powder and monomer [116,117,118]. DLP resolution is determined by the projection plane set by the micromirror device and lens. Resolution is typically in the micron range [115]. According to Saha et al. [119], combining DLP and two-photon lithography (TPL) into a femtosecond projection TPL (FP-TPL) can achieve a higher resolution (sub-500 nm).

One of the main challenges in the production of scaffolds is the occurrence of “debinding failure”. This issue arises when there is a nonuniform removal of the binder from the surface and core of the green body that can be caused by high polymeric content or inadequate powder packing. This often results in the collapse of the printed structure, especially when the granulometry is not optimized [120]. To overcome the problem, researchers have been exploring the use of binders that can contribute to the final glass composition upon firing. A possible solution is silicone. By incorporating silicone as a binder, the production of scaffolds can be improved: such material in fact can facilitate the debinding process and contribute to the overall strength and stability of the final product. It is important to stress that the resulting product of this approach is no longer a composite scaffold but rather a BG scaffold. Elsayed et al. [121] successfully integrated DLP of glass–ceramics with a silicone reactive binder that transformed into silica upon firing. They conducted a comparison between a reference glass scaffold printed with a sacrificial binder and a “silica defective” glass variant (15 wt%) with a silicone binder (H62C). Mechanical tests and SEM images revealed that the silicone component had a binding effect that resulted in homogeneous and crack-free scaffolds with a compressive strength of 3.7 ± 0.4 MPa. In a subsequent study, Dogrul et al. [122] focused on the effect of a new silicone (H44) on a Biosilicate^®^ composite scaffold produced with DLP. Biosilicate^®^ is a BG known for its effectiveness in tissue regeneration that is composed of 23.75Na_2_O–23.75CaO–48.5SiO_2_–4P_2_O_5_ (wt %) [123]. H44 provided a way to fine-tune the microstructures without any cytotoxic effects on stromal cells. Similarly, the use of a silane coupling agent can improve the interaction between the methacrylate polymer matrix and the bioceramic material [124]. This approach was employed by Vyas et al. [125]; in their research, they found that there was a limit on the loading levels of glass particulate for DLP printing. They observed that beyond a certain threshold of 20 wt% of bioactive glass ceramic, the 3D printed parts resulted in uncured or dimensionally inaccurate structures. Despite demonstrating good biocompatibility, the scaffolds produced did not match the mechanical properties of cortical bone even with a lower content of BG. Xu et al. [126] successfully reconstructed a critical-size defect in a rabbit mandible using DLP-printed composite scaffolds. The study introduced a novel BG called AP40mod composed of SiO_2_, P_2_O_5_, CaO, K_2_O, Na_2_O, CaF_2_, and TiO_2_. The scaffolds were seeded with endothelial progenitor cells (EPCs) and bone marrow mesenchymal stem cells (BMSCs), leading to excellent defect repair. However, the use of titanium for fixation necessitated a secondary surgical removal. To address this issue, the authors suggested further investigation into utilizing AP40mod or combinations of high-molecular-weight polymers (e.g., polycaprolactone) to print customized, degradable mesh and staples.

In summary, these studies demonstrated that DLP is a promising method for composite scaffolding due to its versatility and high resolution. However, further research is needed to optimize the mechanical properties that can be achieved through this technique. Moreover, additional in vivo testing is necessary to thoroughly evaluate the effects of various feedstocks on the resulting scaffolds.

## 4. Laser-Based 3D Printing

Selective laser sintering (SLS) is a process that exploits a focused laser beam to rapidly heat and fuse bed powder materials to produce solid parts. There are two primary approaches to SLS: indirect and direct. Indirect SLS involves bonding a mix of ceramic powders and a small amount of polymer binder using a low-power laser scan, resulting in a porous object that may require additional sintering in a furnace for strength. On the other hand, direct SLS employs a high-power laser beam and does not need any polymeric bonding materials, allowing it to produce parts with densities over 80% of the theoretical density [127]. Direct SLS can be further divided into three approaches: solid-state sintering (SSS), liquid-phase sintering (LPS), and selective laser melting (SLM). SSS is a process that produces solid parts by heating a material powder to a temperature just below its melting point [128], resulting in solid bonds between the particles. However, SSS often produces highly porous components [129]. LPS involves heating materials near their melting temperature and using a combination of low- and high-melting-temperature materials to create a dense composite with customized properties. SLM is a laser-based additive manufacturing process that fuses material powders without the use of binding materials, creating fully dense parts with superior mechanical properties. However, SLM parts may suffer some residual stresses that can lead to distortion, cracks, and delamination [127].

The choice between different approaches is not always clear or specified. Therefore, in this section, we will refer to all approaches generically as SLS; however, when possible, we will specify accordingly.

PLA and its stereoisomers such as PLLA and PDLLA are widely used in SLS for TE applications. The production of PDLLA/58S scaffolds for non-load-bearing applications such as the facial skeleton was optimized by Pereira et al. [130] using SLS. To achieve optimal results, the laser power was set at 5.4 W because a lower power failed to promote particle sintering while a higher power led to polymer degradation. The stress–strain curves further revealed that composites with 10 wt% of the BG exhibited the greatest mechanical properties, while a higher BG content resulted in decreased values due to a decrease in the polymer particle coalescence. To improve the biocompatibility of PLLA-based scaffolds and reduce the inflammatory response induced by their degradation, Sun et al. [131] produced PLLA/BG scaffolds doped with DEX. The researchers developed scaffolds with 10 wt% of BG and varying amounts of DEX. Their research confirmed the activity of DEX after printing; the most effective regeneration was seen in vivo for the scaffolds containing the highest concentration of DEX. Notably, the scaffolds doped with DEX exhibited a lower degradation rate compared to pure PLLA scaffolds, likely due to the hydrophobic nature of DEX, which retards water penetration. Shuai et al. [132] investigated the use of SLS-produced mesoporous BG/PGA-PLLA scaffolds doped with varying ratios of Ag^+^ to create an effective antibacterial scaffold. The researchers utilized polydopamine to allow for the adsorption of Ag^+^ and a reduction in well-dispersed metallic nanoparticles in situ. Moreover, the size of the silver nanoparticles could be regulated by adjusting the concentration of Ag^+^ ions and the amount of polydopamine. Their investigation demonstrated a remarkable bacterial inhibition rate of over 99% against Escherichia coli. Additionally, mechanical and in vitro tests confirmed that the scaffolds possessed appropriate properties for bone replacement. However, further research is necessary to evaluate the in vivo response to the Ag content. The limitations of scaffolds—including layer separation and weak interface bonding—have been identified as barriers to effective cell migration and long-term osteochondral repair [133,134]. However, multigraded scaffolds have shown promise in overcoming these challenges [135]. Karl et al. [136] conducted a study on the use of foamed spherical composite particles in SLS to produce multimaterial and multigraded scaffolds for TE. The composite particles consisted of varying amounts of BG content in a PLGA matrix obtained with an approach using a solid in oil in water. However, the researchers encountered issues with larger particles that contained more than 20 wt% of BG because these particles lost their spherical shape and broke during production. As a result, the scaffolds produced in this initial study had poor mechanical properties and were not well suited for TE applications. To address these challenges, the researchers proposed a solution of load-bearing walls made of pure polymer with the composite material being used as a filler phase. This solution aimed for greater accuracy. To achieve this, Karl et al. proposed two technical approaches: point-by-point SLS [137] or using a structure of particles, which has been patented by Aerosint SA (Belgium) [138]. It should be noted that the proposed approaches by Karl et al. have not been implemented or tested yet. Further research and experimentation would be necessary to determine the effectiveness of these solutions.

Another challenge in TE is the degradation rate of scaffolds, which must be carefully controlled to match the growth rate of the tissue being generated. If the scaffold degrades too slowly, it can impede the regeneration of new tissue; if it degrades too quickly, it can compromise the structural integrity of the scaffold and hinder the formation of functional tissue [40]. Borate BGs can create an alkaline environment during their degradation, making it possible to regulate the dissolution rate of composite scaffolds by adjusting the amount of the borate BG. Han et al. [139] investigated this approach and successfully produced and implanted borate BG/PCL scaffolds to treat a critical-size bone defect in a rabbit radius. Using SLS, they were able to optimize both the mechanical properties and the in vitro results of the scaffold; a 20 wt% BG content was chosen as the ideal composition. The scaffolds had a pore size of 650 µm, facilitating good angiogenesis and osteogenesis after 12 weeks in vivo. These promising findings suggested that this composite scaffold holds great potential as a viable option for repairing bone defects.

To summarize, laser-based techniques show great potential for enhancing interface bonding and controlling scaffold-degradation rates. Additionally, the ability to print bioactive molecules using SLS makes it ideal for fabricating drug-delivery scaffolds. However, further optimization is required to achieve mechanical properties suitable for load-bearing scaffolds.

**Figure 2:**
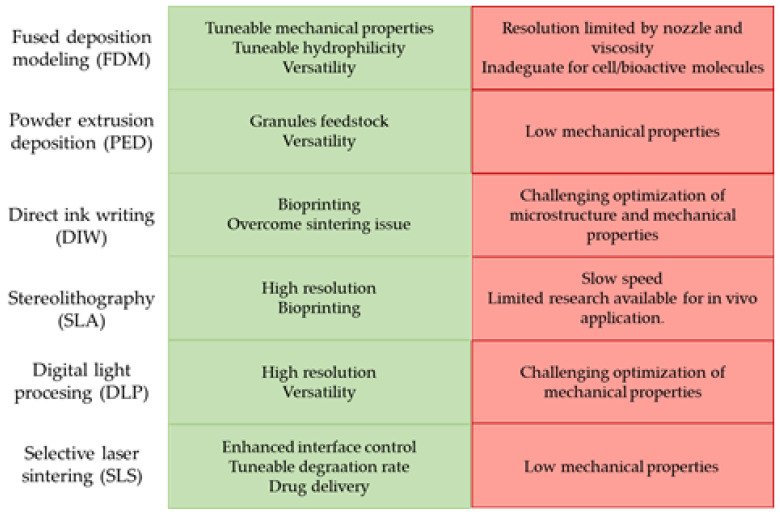
Pros and cons of AM technique for scaffold production.

For a comprehensive list of the techniques and material combinations mentioned in this review, please refer to Table 1. Table 2 provides a brief description of the BGs cited in this review. Additionally, Figure 2 reports a summary of the pros and cons associated with each specific technique discussed.

## 5. Conclusions

In conclusion, the field of TE has made significant progress since its inception. The discovery of BGs and their ability to stimulate the formation of new tissue has opened up a range of possibilities, particularly when combined with polymers to create composites with superior mechanical and biological properties. AM has shown a great potential for scaffold fabrication, enabling the creation of structures with intricate designs and tailored properties for specific tissues. Nonetheless, there are still challenges that need to be addressed such as optimizing scaffold design and fabrication processes and evaluating their long-term safety and efficacy in clinical settings. 

However, the integration of machine learning and artificial intelligence offers promising solutions to these challenges. Machine learning algorithms can analyse vast datasets and unveil intricate patterns, providing valuable insights into the complex interactions between scaffold materials, cell behavior, and tissue regeneration. This integration holds tremendous potential for advancing tissue engineering and optimizing scaffold production. By leveraging machine learning, researchers can expedite the design process, predict scaffold properties, and accelerate the screening of various design possibilities, thus reducing time and costs associated with trial-and-error experiments. Additionally, machine learning can aid in the evaluation of scaffold performance, safety, and personalized treatment recommendations. In summary, the combination of TE, BGs, AM, and machine learning represents a powerful synergy that can revolutionize regenerative medicine. Further research and development are necessary to overcome existing challenges and fully harness the potential of these technologies. By doing so, we can pave the way for innovative approaches that drive tissue engineering to new frontiers, ultimately benefiting patients and transforming healthcare practices.

## Figures and Tables

**Table 1:** List of techniques and material combinations for producing scaffolds via AM.

Technique	Materials	Main Results	Reference
Fused deposition modeling (FDM)	PCL/58S	High tuning of mechanical properties Low hydrophilicity	[61]
	PCL/13-93 PCL/13-93B3	Excellent hydrophilicity Best cell viability for 13-93B3	[65]
	PCL/BG-Ca PCL/BG-Sr PCL/BG-CaCo PCL/BG-SrCo	Increased hydrophilicity after surface treatment Best mechanical properties for BG-SrCo scaffold	[67]
	PCL/BGS-7	Identified a limit on BG content	[68]
	PLA/45S5	Poor bonding between PLA and BG	[58,71]
	SA/13-93	Best mechanical and biological properties for an SA/BG ratio of 4:2	[73]
Powder extrusion deposition (PED)	PCL/BG	Compressive modulus in trabecular bone range	[77]
	PCL/45S5	Best cell activity with 1% BG content	[78]
Direct ink writing (DIW)	Pluronic F-127/PSrBG Pluronic F-127/ICIE16 Pluronic F-127/13-93	Improved sintering of low-silica BG with larger particle size	[82]
	Carboxymethyl cellulose/HSSGG	High compressive strength for Cu-doped BG	[84]
	Carboxymethyl cellulose/45S5	Improved mechanical properties after nanocomposite coating	[87,88]
	Pluronic F-127/47.5B	Failed attempt to produce a porosity gradient	[89]
	PCL-PGS/BG	Elastic modulus in ligament range	[90]
	Ethyl cellulose/13-93 Ethyl cellulose/13-93B3	Superior stability of silicate glass in SBF	[91]
	Pluronic F-127/47.5B	More homogeneous tissue formation of scaffold compared to granules	[92]
	Pluronic F-127/13-93	From brittle to elasto-plastic response after implantation in vivo	[93]
	Chitosan/BG	Positive in vitro results	[94]
	PCL/BG Silk fibroin/BG	Best results for SF/BG scaffold	[98]
	PLA/13-93B3	Death of cells in the bottom layer	[99]
	PVA/BG	Reduced inflammatory response for DEX-doped scaffold	[100,104]
Stereolithography (SLA)	PCL/S53P4	Optimized exposure of BG particles on surface	[110]
	Acrylic resin/BG	Improved stability, mechanical properties, and architecture with PEG-200	[112]
	Acrylic resin/45S5	Optimized BG suspension	[113]
	Acrylic resin/45S5	Increased mechanical properties with partial presintering	[114]
Digital light processing (DLP)	Silicone/WB	Successful production of silica-defective BG scaffold	[121]
	Silicone/biosilicate	Fine-tuned microstructure	[122]
	Acrylic resin/BG	Identified a limit on BG content	[125]
	Acrylic resin/AP40mod	Successful reconstruction in vivo	[126]
Selective laser sintering (SLS)	PDLLA/58S	Optimized the production of scaffold for non-load-bearing application	[130]
	PLLA/BG	Improved regeneration for high content of DEX	[131]
	PGA-PLLA/mesoporous BG	Antibacterial scaffold with good mechanical properties	[132]
	PLGA/45S5	Produced multigraded scaffold with poor mechanical properties	[136]
	PCL/borate BG	Optimized the process for borate BG	[139]

**Table 2:** List of bioactive glasses and their main characteristics.

Bioactive Glass	Characteristics	Reference
45S5	Silicate Best biological properties	[24,29]
S53P4	Silicate Antibacterial properties	[111]
13-93	Silicate Large working range	[30]
13-93B3	Borate High degradation rate	[66]
58S	Silicate Alkali-free Sol–gel	[62]
ICIE16	Mixed alkali silicate glass	[31]
HSSGG	High silica content Sol–gel	[84]
47.5B	Silicate Large working range	[89,92]
WB	Borosilicate	[140]
Biosilicate	Silicate Fair machinability	[123]
AP40mod	Silicate TiO_2_-containing	[126]
BGMS10	MgO- and SrO-containing Ultrahigh crystallization temperature	[32]

## Data Availability

Not applicable.

## Abbreviations

µ-CT	Micro-computed tomography
AM	Additive manufacturing
BG	Bioactive glass
BMSC	Bone marrow mesenchymal stem cell
DEX	Dexamethasone
DIW	Direct ink writing
DLP	Digital light processing
DMD	Digital micromirror device
ECM	Extracellular matrix
EPC	Endothelial progenitor cell
FDM	Fused deposition modeling
FP-TPL	Femtosecond projection TPL
HA	Hydroxyapatite
HSSGG	High-silica sol–gel glass
LPS	Liquid-phase sintering
NIR	Near-infrared
PED	Powder extrusion deposition
PI	Photoinitiator
rBMSC	Rat bone mesenchymal stem cell
RM	Regenerative medicine
SA	Sodium alginate
SBF	Simulated body fluid
SLA	Stereolithography
SLM	Selective laser melting
SLS	Selective laser sintering
SSS	Solid-state sintering
TE	Tissue engineering
TPL	Two-photon lithography
XRD	X-ray diffraction
